# Prefrontal cortex infusion of beta‐hydroxybutyrate, an endogenous NLRP3 inflammasome inhibitor, produces antidepressant‐like effects in a rodent model of depression

**DOI:** 10.1002/npr2.12099

**Published:** 2020-03-03

**Authors:** Naofumi Kajitani, Masaaki Iwata, Akihiko Miura, Kyohei Tsunetomi, Takehiko Yamanashi, Ryoichi Matsuo, Tsuyoshi Nishiguchi, Saki Fukuda, Mayu Nagata, Midori Shibushita, Takahira Yamauchi, Shenghong Pu, Yukihiko Shirayama, Ken Watanabe, Koichi Kaneko

**Affiliations:** ^1^ Department of Neuropsychiatry Faculty of Medicine Tottori University Yonago Japan; ^2^ Department of Psychiatry Nara Medical University School of Medicine Kashihara Japan; ^3^ Department of Psychiatry Teikyo University Chiba Medical Center Ichihara Japan; ^4^ Watanabe Hospital Tottori Japan

**Keywords:** beta‐hydroxybutyrate, depression, inflammasome, NLRP3, prefrontal cortex, stress

## Abstract

**Aims:**

Neuroinflammation is deeply related to the pathophysiology of depression. Beta‐hydroxybutyrate (BHB), which is an endogenous ketone body, exerts anti‐inflammatory effects, and peripheral administration of BHB induces antidepressant effects in an animal model of depression; however, it is unclear whether BHB specifically mediates these actions in the brain. Thus, we administered BHB directly into the brain in a rodent model of depression using a chronic unpredictable stress (CUS) paradigm.

**Methods:**

BHB was continuously microinjected into the prefrontal cortex (PFC) using osmotic pumps for 21 days. Behavioral testing included the forced swim test (FST) and the open field test (OFT); the levels of pro‐inflammatory cytokines, such as interleukin 1β (IL‐1β) and tumor necrosis factor α (TNF‐α), were quantified in the PFC, and the concentration of corticosterone in blood serum was measured.

**Results:**

BHB administration into the PFC significantly decreased immobility time in the FST, without significantly altering locomotor activity assessed in the OFT. Also, CUS significantly increased the levels of TNF‐α in the PFC and decreased serum corticosterone levels; these changes were attenuated by BHB administration. These findings suggest that a small amount of BHB administered into the PFC directly produces antidepressant effects, possibly through anti‐inflammatory mechanisms, and can improve hypothalamus‐pituitary‐adrenal axis responses.

**Conclusion:**

BHB may be a novel therapeutic candidate for the treatment of depression based on the neuro‐inflammatory hypothesis, and the PFC is a region implicated in the antidepressant action of BHB.

## Abstract 2

A small amount of beta‐hydroxybutyrate administered into the prefrontal cortex directly produces antidepressant effects, possibly through anti‐inflammatory mechanisms, and can improve hypothalamus‐pituitary‐adrenal axis responses.

## Graphical abstract

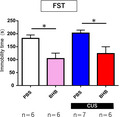

## 1 INTRODUCTION

Depression is a mental illness involving symptoms of depressed mood and diminished interest or pleasure. Although the pathophysiology of depression is still unclear, stressful life events can be risk factors that induce neuroplastic changes in the brain, such as decreased neurogenesis in the hippocampus and a loss of dendritic spine density 1, 2, 3. Current antidepressant treatment based on the monoamine hypothesis produces inadequate improvement in 1/3 of depressive patients 4; thus, the identification of alternative mechanisms of depression is required to develop novel therapeutic strategies. It is well known that depression often coexists with chronic diseases such as diabetes, cancer, heart disease, and rheumatoid arthritis, and these diseases share a feature of chronic inflammation 5, 6, suggesting that immune reactivity and inflammatory processes may be common factors underlying the neurophysiological changes associated with depression. Recent studies have shown a relationship between brain inflammation and depression, as administration of interleukin‐1β (IL‐1β), a pro‐inflammatory cytokine, in the brain can cause depressive‐like behavior 7. In humans, increases in inflammatory cytokine expression, including IL‐1β and IL‐18, in the peripheral blood of depressed patients have been reported; these expression levels become normalized with recovery from depression 8, 9. It has also been shown that nucleotide‐binding domain, leucine‐rich repeat and pyrin domain containing 3 (NLRP3), a pattern recognition receptor involved in the production of IL‐1β and IL‐18, are increased in serum; again, these changes can be normalized by treatment with tricyclic antidepressants 10. NLRP3 reacts to various danger signals to form a protein complex called an inflammasome, which generates inflammatory cytokines through the activation of caspase‐1. Our previous study demonstrated that stress increases adenosine tri‐phosphate (ATP) production, which acts as a danger signal in the prefrontal cortex (PFC) and hippocampus. Furthermore, the activation of NLRP3 and the increase of IL‐1β and tumor necrosis factor α (TNF‐α) in the hippocampus are induced by stress 11, while inhibition of NLRP3 inflammasome activity by ATP receptor antagonists, purinergic Type 2X7 receptor (P2X7R) antagonists, or the neutralization of IL‐1β by an IL‐1β antibody improves depressive‐like behaviors 11. Thus, inhibiting ATP‐P2X7R‐NLRP3 signaling is a possible strategy for treating depression induced by stress.

Beta‐hydroxybutyrate (BHB) is an endogenous ketone body that supports mammalian cell metabolism during states of energy deficiency, such as those induced by fasting or exercise 12, 13. In recent years, several reports have shown that BHB may have an antidepressant effect 14, 15, likely by inhibiting the activation of the NLRP3 inflammasome 16. In addition, our previous study showed that peripheral administration of BHB decreased inflammatory cytokine concentrations in the hippocampus, such as IL‐1β and TNF‐α, and improved depressive and anxiety‐like behaviors in a rodent model of chronic unpredictable stress (CUS) 15. While BHB is thought to have potential antidepressant actions, it is not known whether peripherally administered BHB can act directly in the brain or if its effects are mediated strictly through peripheral mechanisms. Therefore, in this study, we aimed to investigate whether direct, local infusion of BHB into the brain is effective in alleviating stress‐induced depressive‐like behaviors.

Although the specific brain regions associated with depression have not been identified, human studies have shown certain morphological changes, such as hippocampal atrophy^17^, ^18^ and blood flow reduction in the frontal lobe 19. In animal stress models, an increase in inflammatory cytokine concentration in the PFC and hippocampus has been observed, and activation of the NLRP3 inflammasome occurs in the PFC 20, 21, 22. Recently, it has been reported that PFC administration of ketamine produces rapid and potent antidepressant actions 23. Because inflammation in the PFC may be related to the pathophysiology of depression, and administration of ketamine in the PFC induces an antidepressant effect, we aimed to confirm whether the antidepressant action of BHB can be mediated through direct administration into the PFC in CUS model rats.

## 2 MATERIALS AND METHODS

The experimental procedures were conducted in accordance with the Institutional Animal Care Guidelines and were approved by the Tottori University Animal Care and Use Committee (Approval number h31‐Y012). Efforts were made to minimize animal suffering.

### 2.1 Animals

Male Sprague‐Dawley rats, 7‐8 weeks of age, were obtained from Charles River Laboratories. Two rats were housed per cage on a 12 h/12 h light/dark cycle (lights on at 7:30 AM). Temperature was maintained at 25°C, and food and water were freely available. One week before the experimental procedures, rats were acclimatized to the laboratory. Their mean body weight was about 350 g before the start of experiments.

### 2.2 Reagents

DL‐BHB (Tokyo Chemical Industry) was dissolved in phosphate‐buffered saline (PBS) (NaCl 137 mM, KCl 2.7 mM, Na_2_HPO_4_.12H_2_O 8.1 mM, KH_2_PO_4 _1.47 mM). BHB was prepared at a concentration of 80 mg/mL based on a previous report 24. Both BHB and PBS solutions were adjusted to pH 7.3.

### 2.3 Cannulation surgery

**Figure 1:**
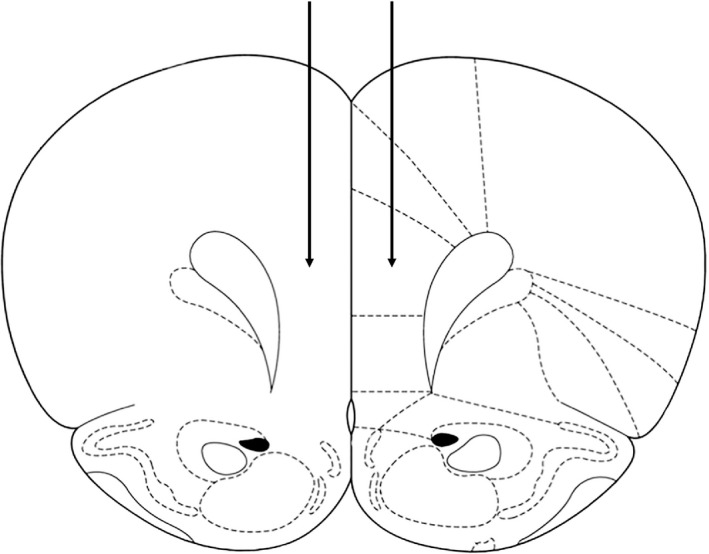
Surgical cannulation. The cannulas (4 mm in length) were inserted into the PFC (coordinates: anteroposterior + 3.2 mm, dorsolateral ± 0.6 mm from bregma, ventral 4.0 mm from the skull surface). Each arm of the cannula was connected to an osmotic pump (nominal pumping rate: 0.15 μL/h; duration: six weeks). The pumps were filled with PBS or BHB solutions. PFC: prefrontal cortex; PBS: phosphate‐buffered saline; BHB: beta‐hydroxybutyrate

Animals were anesthetized with an intramuscular (im) injection of ketamine/xylazine (80/6 mg/kg) and held in a stereotactic frame. Cannulas were inserted into the PFC (coordinates: anteroposterior + 3.2 mm, dorsolateral ± 0.6 mm from bregma, ventral 4.0 mm from the skull surface) (Figure 1). The cannulas were homemade, with a length of 4 mm. Each arm of the cannula was connected to an osmotic pump (ALZET® Osmotic Pumps Model 2006. Nominal pumping rate: 0.15 μL/h; duration: six weeks). One pump was used for each cannula (two pumps in total per rat). The pumps were filled with PBS or BHB solutions. Antibacterial penicillin G (6000 units) (Meiji Seika Pharma Co., Ltd.) was injected before surgery, and an analgesic (carprofen, 5 mg/kg; Zoetis Japan Co., Ltd.) was administered for three days after surgery. Rats were allowed a recovery period of one week after surgery before starting the CUS paradigm.

### 2.4 Experimental groups

**Figure 2:**
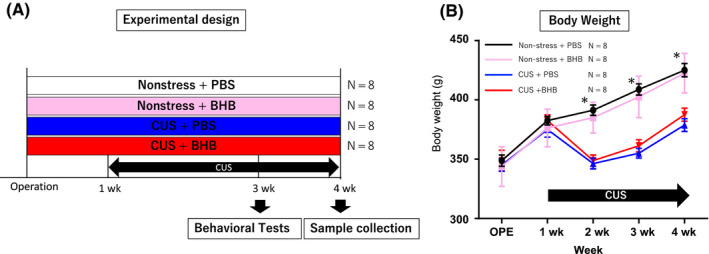
Experimental design and body weight tracking. (A) Schematic of the experimental design. Rats were divided into four groups: (1) nonstress + PBS, (2) nonstress + BHB, (3) CUS + PBS, (4) CUS + BHB. Behavioral tests were conducted two to three weeks after the beginning of the stress paradigm; brain and blood samples were collected after all behavioral tests were completed. (B) Body weight tracking. Compared to the nonstress group, the CUS group started to lose weight one week later and then gained weight. However, the nonstress group and the CUS group continued to exhibit differences in body weight. *P* < .001 for comparisons between nonstress + PBS vs CUS + PBS, nonstress + PBS vs CUS + BHB, nonstress + BHB vs CUS + PBS, and nonstress + BHB vs CUS + BHB. Error bars represent the standard error of the mean (SEM). PBS: phosphate‐buffered saline; BHB: beta‐hydroxybutyrate; CUS: chronic unpredictable stress

Rats were divided into four groups: (a) nonstress + PBS, (b) nonstress + BHB, (c) CUS + PBS, (d) CUS + BHB. Behavioral tests were conducted two to three weeks after the start of the CUS paradigm. Blood samples were collected after all behavioral tests were completed (Figure 2A).

### 2.5 Chronic unpredictable stress (CUS)

The CUS paradigm is a rodent model of depression in which rats are exposed to a variable sequence of two mild and unpredictable stressors per day, preventing habituation, as previously described 15. The stressors included cage tilt (tilting the cage 45°, overnight), crowding (ten animals per cage), cold temperature exposure (one hour at 4°C), immobilization in tapered plastic film tubes (one hour), exposure to wet bedding (wetting the floor overnight or during the daytime), no bedding (removal of wood chips overnight or during the daytime), food deprivation (overnight), water deprivation (overnight), isolation (overnight), cage rotation (one hour), and lights off during the daytime or lights on overnight. These stressors were adapted from our previous study 15. Control rats were handled daily.

### 2.6 Behavioral tests

#### 2.6.1 Forced swim test (FST)

Rats were placed for five minutes in a plastic bucket filled with water (24°C, 34 cm depth). Immobility was defined as the point at which the rat ceased struggling and stayed in the water without movement, except those necessary to keep their heads above water. Tests were recorded and scored by a blinded observer. The water was changed after each trial.

#### 2.6.2 Open field test (OFT)

Rats were placed in an open field apparatus (90 cm × 90 cm × 45 cm) and allowed to explore for ten minutes. The test was conducted in a bright environment. Locomotor activity (total distance travelled) was assessed using TopScan Suite (CleverSys, Inc). The open field apparatus was cleaned after each trial.

### 2.7 Western blotting

Western blotting was conducted as previously described 15. Briefly, the frozen PFC tissue samples were homogenized in ice‐cold buffer. The protein samples were separated using 12% sodium dodecyl sulfate‐polyacrylamide gel electrophoresis (SDS‐PAGE), then transferred to nitrocellulose membranes. The membranes were incubated with primary antibodies for IL‐1β (ab9722; 1:2,500 dilution, ie, 0.2 μg/mL, Abcam) and TNF‐α (AB1837P; 1:2,500, ie 0.2 μg/mL, Merck Millipore), followed by incubation with an anti‐rabbit secondary antibody (ab16284; 1:10,000 dilution, ie 0.1 μg/mL, Abcam). Densitometric analysis of Western blot bands was performed using ImageJ software, version 1.51 (National Institutes of Health).

### 2.8 Serum corticosterone measurement

After the behavioral tests were completed, blood was collected (5:00 PM‐6:00 PM) from the site of decapitation and stored overnight at 4°C. The next day, samples were centrifuged at 500 *g* for 1 min at 4°C to remove debris, the supernatant was collected, and serum corticosterone levels were measured by a chemiluminescent enzyme immunoassay (CLEIA) method based on the manufacturer's instructions (Roche Diagnostics K.K).

### 2.9 Statistical analyses

All statistical analyses were conducted using Statistical Package for the Social Sciences (SPSS) Statistics 19.0. For comparisons among the four groups, analyses of variance were conducted, followed by Tukey's post hoc tests. The data are presented as means ± standard error of the mean (SEM). *P*‐values < .05 were considered statistically significant.

## 3 RESULTS

### 3.1 Rats exposed to CUS displayed weight loss

We examined the influence of the CUS paradigm on rats’ body weight (BW). At the beginning of the experiment, there were no significant differences in BW among the four groups (*F*_(3,31) _= 0.162, *P* = .921, n = 8). Compared with the nonstress group, the CUS group lost weight during the first week after starting the CUS paradigm. After that initial loss, the nonstress and CUS groups gained weight in parallel, but continued to show significant differences in BW (Figure 2B). This result is similar to those observed in our previous study 15.

### 3.2 BHB microinjection into the PFC induced antidepressant effects in the FST

**Figure 3:**
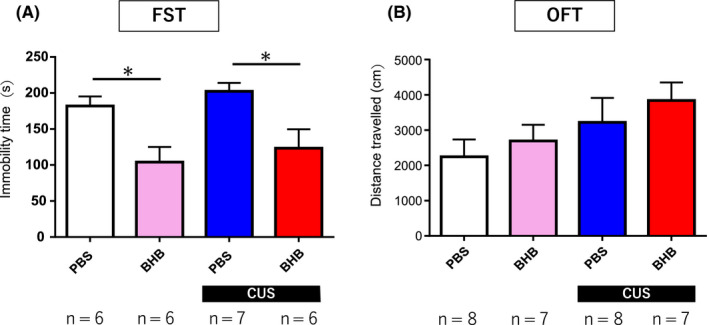
Forced swim test and assessment of locomotor activity. (A) Forced swim test. Although CUS did not induce stress responses compared with nonstress controls, BHB administration shortened the immobility time in both the nonstress and the CUS group. *P* < .05 for comparisons between nonstress + PBS vs nonstress + BHB and CUS + PBS vs CUS + BHB. (B) Open field test. The OFT was conducted to determine whether locomotor activity correlated with the changes in immobility time observed in the FST. There was no significant difference between the groups. Error bars represent the standard error of the mean (SEM). FST, forced swim test; CUS: chronic unpredictable stress; BHB: beta‐hydroxybutyrate; PBS: phosphate‐buffered saline; OFT, open field test

Previously, we demonstrated an antidepressant effect in the FST following peripheral administration of BHB; however, it was unclear whether this resulted from a direct effect in the central nervous system (CNS). In order to assess whether the effects were direct, we conducted the FST following cannulation and microinjection of BHB into the PFC. Compared to the nonstress + PBS group, the CUS + PBS group showed no difference in immobility time (*P* = .857). However, in both the nonstress and the CUS groups, BHB administration into the PFC resulted in significantly shorter immobility times (*F*_(3,24) _= 6.484, *P* < .05, n = 6‐7) (Figure 3A). The results demonstrate that BHB exerts antidepressant effects by directly acting in the CNS when administered in the PFC, confirming our hypothesis.

### 3.3 No difference in locomotor activity was observed

The OFT was performed to determine whether changes in locomotor activity correlated with changes in immobility time observed in the FST. Locomotor activity was increased in the CUS group compared with the nonstress group. In addition, locomotor activity was increased following BHB administration; however, there was no significant difference between the treated CUS and no‐stress groups Although there was a small but nonsignificant difference in the OFT, the results were not consistent with the differences in immobility times observed in the FST (*F*_(3,29) _= 1.516, *P* = .234, n = 7‐8) (Figure 3B).

### 3.4 PFC BHB administration attenuated the increase in TNF‐α induced by CUS

**Figure 4:**
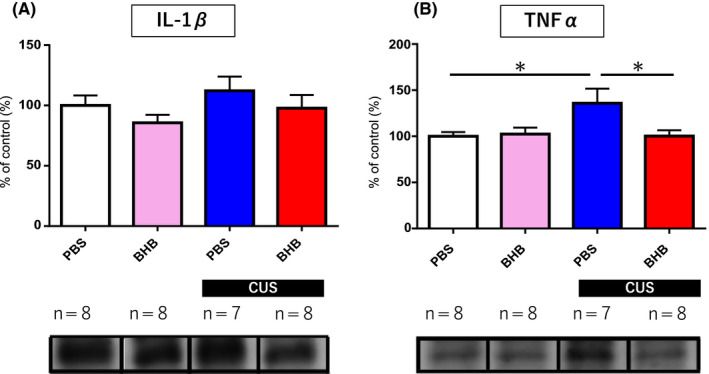
Pro‐inflammatory cytokine levels in the PFC. (A) Comparison of IL‐1β levels. Neither CUS nor BHB affected IL‐1β levels in the PFC. (B) CUS increased TNF‐α levels in the PFC, and this change was completely attenuated by BHB administration into the PFC. Error bars represent the standard error of the mean (SEM). *P < .05. PFC, prefrontal cortex; IL‐1β, interleukin 1‐beta; CUS: chronic unpredictable stress; TNF‐α, tumor necrosis factor‐alpha; BHB: beta‐hydroxybutyrate. PBS: phosphate‐buffered saline

To determine whether stress affects inflammatory cytokine levels in the brain and whether intracerebral administration of BHB has any effect on these cytokine levels, Western blots of PFC samples were performed. The levels of TNF‐α in the PFC were significantly increased in rats exposed to CUS compared with nonstressed control rats (*P* < .05). In addition, BHB administration into the PFC significantly attenuated the upregulation in TNF‐α levels induced by CUS (*P* < .05) (Figure 4B). On the other hand, we found no significant change in IL‐1β levels in the PFC (*F*_(3,30) _= 1.277, *P* = .302, n = 7‐8) (Figure 4A).

### 3.5 CUS changed the hypothalamus‐pituitary‐adrenal (HPA) axis response, which was attenuated by BHB administration

**Figure 5:**
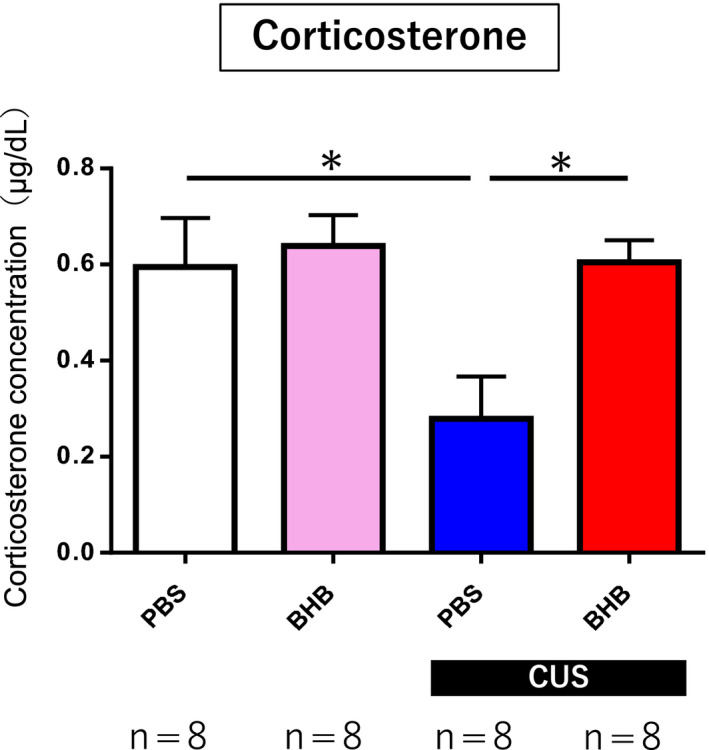
Serum corticosterone measurement. Blood samples were collected after all behavioral tests were completed to measure serum corticosterone levels. Serum corticosterone levels decreased significantly following CUS exposure. This change was attenuated by BHB administration. *P* < .05 for comparisons between nonstress + PBS vs CUS + PBS and CUS + PBS vs CUS + BHB. Error bars represent the standard error of the mean (SEM). CUS: chronic unpredictable stress; BHB: beta‐hydroxybutyrate; PBS: phosphate‐buffered saline

Stress responses can also be evaluated by quantifying HPA axis activity inferred by the levels of corticosterone in the blood. In this study, serum corticosterone levels were significantly reduced following CUS exposure compared with nonstressed control rats; this change was completely attenuated by BHB administration into the PFC (*F*_(3.31) _= 4.655 *P* < .05 n = 8) (Figure 5). In general, stress initiates adaptive processes, such as corticosterone secretion, that allow the organism to physiologically respond to threats; however, long‐term and cumulative stress is known to reduce or dysregulate HPA axis activity to limit the deleterious effects of prolonged corticosterone secretion. In this study, the decreased levels of corticosterone in serum caused by CUS were attenuated by the administration of BHB, possibly through reduction of stress responses.

## 4 DISCUSSION

Inflammation is now known to be involved in the pathophysiology of depression, and BHB administration can induce antidepressant effects by suppressing NLRP3, a critical complex that senses stress molecules and mediates inflammatory responses. Although BHB reportedly exerts antidepressant effects following peripheral administration, it was not previously clear whether it directly acted in the brain. In this study, we administered BHB directly into the PFC to exclude the possibility of peripheral action. We showed that depressive behavior in the FST was improved by the direct administration of BHB into the PFC. In addition, the levels of TNF‐α, a pro‐inflammatory cytokine that is upregulated in the PFC by CUS, were reduced by direct administration of BHB. Also, impaired responsiveness of the HPA axis induced by CUS was attenuated by BHB administration. These results indicate that the antidepressant effects of BHB can be mediated directly in the brain, likely through anti‐inflammatory mechanisms, and that the PFC is at least partially responsible for mediating BHB’s effects in the CNS.

Inflammatory cytokines, such as IL‐1β, are implicated in the pathophysiology of depression. NLRP3, a complex of pattern recognition receptors, and its active form, the inflammasome play critical roles in activating IL‐1β and IL‐18, and have been implicated in multiple chronic inflammatory diseases, such as infectious diseases, autoimmune diseases, diabetes, Alzheimer's disease, and arteriosclerosis, all of which are common comorbidities of depression 25, 26, 27, 28. Thus, NLRP3 may be a key component that mediates inflammation in depression. NLRP3 is expressed mainly in microglia and astrocytes in the brain 29; it detects various dangerous substances and initiates inflammatory responses. When NLRP3 forms an inflammasome, procaspase‐1 is converted to mature caspase‐1, which, in turn, leads to the maturation of pro‐IL‐1β to IL‐1β. The presence of IL‐1β in the brain is associated with depressive behavior 7, and inhibition of the NLRP3 inflammasome or suppression of inflammatory cytokine expression reduces depressive‐like behaviors 11, 15, 30.

BHB is a ketone body synthesized in the liver of mammals that is used as an alternative energy source in the brain in states of glucose deprivation. BHB has anti‐inflammatory 31 and neuroprotective effects and may have therapeutic potential in the treatment of degenerative diseases such as Huntington's disease, Parkinson's disease, and Alzheimer's disease 32, 33, 34; thus, BHB is thought to affect the CNS. There is evidence that a ketogenic diet, which leads to elevated levels of BHB in the serum and brain, produces antidepressant effects 35, as seven days on a ketogenic diet increases the levels of serum BHB and decreases immobility time in the FST 36. In addition, adult offspring previously exposed to a prenatal ketogenic diet exhibits reductions in anxiety‐like behaviors in the OFT and depressive‐like behaviors in the FST. In that study, many neuroanatomical differences were shown to exist between control animals and the offspring of those exposed to a ketogenic diet, including volumetric enlargement of the cerebellum, and reductions in the hypothalamus and corpus callosum 35. Recent reports have shown that BHB itself also induces antidepressant effects in animals 14, 15. In our previous study, we confirmed that chronic peripheral administration of BHB decreased depressive‐like behavior caused by CUS, and that BHB administration suppressed the increase in inflammatory cytokine expression induced by acute stress in this model 15. Another group showed that three weeks of repeated intraperitoneal BHB administration ameliorated depressive behaviors in a mouse model of depression involving spatial restraint stress and dexamethasone administration, as BHB administration increased histone3‐lysine9‐β‐hydroxybutyrylation, leading to upregulated expression of brain‐derived neurotrophic factor (BDNF) 14.

BHB is transported into the brain via a monocarboxylate transporter expressed at the blood‐brain barrier, and it has already been confirmed that peripheral administration leads to an increased BHB concentration in the brain 15, 37. However, the rate at which BHB is transported to the brain and the kinetics of its distribution to various regions are not clear. In addition, because inflammatory cytokines are present in the periphery and can affect the brain through several mechanisms 38, BHB administered peripherally may act by reducing inflammatory cytokines outside the CNS. Recently, it has been shown that peripheral inflammatory mediators can be transmitted to the brain via the afferent vagus nerve, and the brain modulates peripheral inflammation via the efferent vagus nerve 39. Evidence that peripheral inflammation can affect the brain is increasing, and it is important to clarify whether substances that modulate anti‐inflammatory effects can act directly in the brain to reduce depressive‐like symptoms. If the anti‐inflammatory effect of BHB is mediated in the brain to cause an antidepressant response, it was hypothesized that intracerebral administration would result in antidepressant effects, which we confirmed in this study.

Here, BHB was administered into the prelimbic region of the PFC. Morphological changes in the PFC of depressed patients include decreased volume and decreased blood flow 3, 19. Postmortem analysis of the PFC of suicide victims has shown elevated levels of inflammatory cytokines such as IL‐1β, IL‐6, and TNF‐α 40, and suicide victims that have suffered from depression and schizophrenia exhibit increased microglial reactivity 40. Increased microglial activation and inflammatory cytokine expression in the PFC have been linked to psychiatric disorders. In animal experiments, dendritic atrophy occurs in the PFC in response to stress 41, 42. There are also reports of increased NLRP3 inflammasome activity and elevated inflammatory cytokine levels, such as IL‐1β, in the PFC after exposure to stress 20. Antidepressant effects are also observed following administration of ketamine in the PFC 43, and reports that have confirmed the reduction of stress‐induced inflammatory cytokines and depressive‐like behavior due to the lack of toll‐like receptor (TLR) in the PFC 44 suggest that the PFC may be a an important target for antidepressant action.

The CUS paradigm employed in this study is often used as a depression model with high face validity, construct validity, and predictive validity 45, 46, 47; however, we could not confirm the depression‐inducing effect of the CUS paradigm, especially in the behavioral testing, possibly because intracerebral injection is highly invasive and the surgery itself may have induced stress responses. Also, because the reliable operation period of the osmotic pumps we used was only six weeks, we shortened the CUS paradigm to three weeks, which may partially explain the ineffectiveness of the CUS model. Despite such limitations, BHB showed a strong antidepressant effect in the FST in this study. The OFT was also performed to rule out the effects of increased locomotor activity, and there were no significant differences between groups.

In addition to the behavioral tests, changes in pro‐inflammatory cytokines and corticosterone levels were examined in vivo. In this study, CUS did not affect IL‐1β levels, but induced a significant increase in TNF‐α levels. On the other hand, BHB attenuated the upregulation of TNF‐α. The fact that we observed changes in TNF‐α and no effects on IL‐1β levels are consistent with the results reported in our previous studies 11, 15. While IL‐1β levels typically increase in response to stress, the change is transient, as compensatory mechanisms quickly restore the concentration to basal levels. On the other hand, since TNF‐α levels continue to change long after the initial insult, the differences are relatively easier to observe. In this study, while IL‐1β levels tended to increase slightly in response to stress and decreased following BHB administration, no significant differences were observed. Previous studies have reported that P2X7R inhibitors, which can regulate the ATP‐NLRP3 pathway, suppress TNF‐α upregulation. Changes in TNF‐α levels are thought to be downstream of IL‐1β and may be controlled by inhibition of the ATP‐NLPR3 pathway. Thus, it is assumed that stress increases TNF‐α and, possibly to a lesser extent, IL‐1β levels in the PFC and can cause neuropathy, but BHB suppresses neuroinflammation and exerts a neuroprotective effect.

The PFC is rich in glucocorticoid receptors 48, 49, and it is thought that the PFC is involved in the regulation of the HPA axis, along with the hippocampus 41, 50. Chronic stress has been shown to reduce the expression levels of glucocorticoid receptors in both the PFC and the hippocampus 51, 52, and deficiency of glucocorticoid receptors in the PFC is reported to prolong immobility time in the FST 53, 54. In some chronic stress models in animals, it has also been reported that HPA activity stops increasing over time 55. In our study, the CUS group exhibited significantly lower corticosterone levels compared with the nonstress group, while BHB administration attenuated this decrease in corticosterone production, even under the stress condition. This suggests that BHB may suppress the alterations in corticosterone receptor expression in the PFC caused by stress and may help maintain HPA system function.

There are several methodological limitations to this study. We cannot fully claim that improvements in abnormal cytokine levels and HPA dysfunction are responsible for the antidepressant effects of BHB because it is not clear whether these changes are the main contributors to the antidepressant effect or just a consequence of other mechanisms of change; further study is required to determine causal relationships. Also, a recent study reported that immobility in the FST may not only be related to “despair,” but may also be interpreted as an “adaptive learned response” 56. This is a very important distinction, and we need to cautiously interpret the results of the FST.

Due to the operating period of the osmotic pump mentioned above, the duration of the CUS paradigm might be inadequate. Stress other than that caused by the CUS paradigm, such as surgery, postoperative pain, and the continuous administration of fluids could have influenced the results. Because BHB was administered to a specific brain region such as the PFC, other sites related to the pathophysiology of depression such as the hippocampus, amygdala, and nucleus accumbens should also be evaluated. Also, alternate pathways mediated by hydroxycarboxylic acid receptor 2 (HCAR2) and histone deacetylase (HDAC) are reportedly involved in the mechanistic action of BHB 31. In order to elucidate BHB’s mechanisms through alternate signaling pathways, further experiments would be necessary.

In conclusion, direct administration of BHB into the PFC improved CUS‐induced depressive‐like behaviors and normalized altered inflammatory and corticosterone‐related responses. The antidepressant action of BHB is observed even if it is infused into a specific brain region, such as the PFC, suggesting that BHB administration may be a useful approach to target the neuro‐inflammatory mechanisms associated with depression.

## CONFLICT OF INTEREST

The authors declare that they have no conflicts of interest relevant to the content of the article.

## DATA REPOSITORY

Raw data are shown in Table S1.

## APPROVAL OF THE RESEARCH PROTOCOL BY AN INSTITUTIONAL REVIEW BOARD

n/a.

## INFORMED CONSENT

n/a.

## REGISTRY AND THE REGISTRATION NO. OF THE STUDY/TRIAL

n/a.

## ANIMAL STUDIES

The experimental procedures were conducted in accordance with the Institutional Animal Care Guidelines and were approved by the Tottori University Animal Care and Use Committee (Approval number h31‐Y012). Efforts were made to minimize animal suffering.
